# Age at Menarche and Menopause, Reproductive Lifespan, and Risk of Cardiovascular Events Among Chinese Postmenopausal Women: Results From a Large National Representative Cohort Study

**DOI:** 10.3389/fcvm.2022.870360

**Published:** 2022-09-09

**Authors:** Lu Chen, Zhen Hu, Xin Wang, Yuxin Song, Zuo Chen, Linfeng Zhang, Congyi Zheng, Jillian Vallis, Haoqi Zhou, Xue Cao, Yixin Tian, Jiayin Cai, Runqing Gu, Yilin Huang, Zengwu Wang

**Affiliations:** ^1^Division of Prevention and Community Health, National Center for Cardiovascular Disease, National Clinical Research Center of Cardiovascular Disease, State Key Laboratory of Cardiovascular Disease, Fuwai Hospital, Peking Union Medical College and Chinese Academy of Medical Sciences, Beijing, China; ^2^PGY3 General Surgery, Memorial University of Newfoundland, St. John’s, NL, Canada

**Keywords:** age at menarche, age at menopause, reproductive lifespan, cardiovascular events, postmenopausal women

## Abstract

**Background:**

At present, the association between age at menarche and menopause, reproductive lifespan, and cardiovascular disease (CVD) risk among Chinese postmenopausal women is not clear, and some related researches are contradictory.

**Methods:**

A total of 6,198 Chinese postmenopausal women with a mean age of 63.6 years were enrolled at baseline in 2012–2015 and followed up for 5 years. A standardized questionnaire was used to collect relevant information by well-trained interviewers. Physical examination of the participants was performed by trained medical staff. CVD events were observed during follow-up. Cox proportional hazards models were used to estimate hazard ratios between reproductive characteristics and CVD events.

**Results:**

Age at menarche was positively associated with CVD events (HR, 1.106; 95%CI, 1.047–1.167). There was a negative association between age at menopause and CVD risk in postmenopausal women with comorbidity (HR, 0.952; 95%CI, 0.909–0.996). Reproductive lifespan was negatively associated with CVD events (HR, 0.938; 95%CI, 0.880–0.999). The CVD risk increased by 10.6% for every 1-year increase in age at menarche. The CVD risk reduced by 6.2% for every 1-year increase in age at menopause in women with comorbidity. The CVD risk reduced by 3.8% for every 1-year increase in reproductive lifespan.

**Conclusions:**

Based on the large prospective study with a nationally representative sample, Chinese postmenopausal women with late age at menarche and shorter reproductive lifespan have higher risk of CVD events.

## Introduction

Cardiovascular disease (CVD) is the dominant cause of death throughout the world (1), accounting for 32% of the total deaths (2). In China, CVD is the leading cause of death and disability (1), accounting for approximately 45% of all deaths (3). Meanwhile, CVD as the principle cause of death in women is still under-recognized and undertreated (4). Therefore, comprehensive recognition of CVD risk factors and timely implementation of appropriate interventions for women is of tremendous public health importance.

Menarche is a marker of puberty and the onset of ovarian and other endocrine functions relating to reproduction (5). The results of previous studies on the relationship between age at menarche, menopause, reproductive lifespan, and the risk of CVD are inconsistent (6–11). For example, a systematic review revealed that three out of four studies show a generally linear association between a decrease in age at menarche with an increase in CVD risk (11). However, the UK Million Women Study manifested a significant U-shaped relationship between age at menarche and coronary heart disease (CHD) risk (6). Data from Asian populations were also contradictory and inconclusive (11).

Age at onset of menopause may be a marker for not only reproductive aging but also for general health and somatic aging (12). Adverse changes in CVD risk factors occur around the menopausal transition (13–15). A meta-analysis including 50,000 Western women showed a negative linear association between age at menopause and CVD (16). A follow-up study found a non-significant increased risk of death caused by CHD and stroke among women with earlier natural menopause (12). This discrepancy may be due in part to a variety of confounding variables including genetic background, race, study design, or environmental factors amongst different populations.

The interval between menarche and menopause defines a woman’s natural reproductive span. Due to exposure to different hormonal levels, early or late onset of these events may be associated with increased risk of many chronic health problems. However, the association between duration of reproductive lifespan and CVD risk has not been investigated thoroughly. A shorter duration of reproductive life span was associated with a higher Framingham Risk Score. However, this study was limited by its cross-sectional design to investigate a long-term CVD event risk (17). Results from the Australian Longitudinal Study of Women’s Health showed that an increase in reproductive lifespan per year was associated with a 7% reduction in stroke risk, but this association disappeared when only the duration of endogenous estrogen exposure was considered (18). Further, the association was either null or mixed (19, 20). These findings suggest that the association between reproductive lifespan and CVD is not completely clear and requires further investigation.

Most previous studies addressing this objective are conducted in Western populations. Asian women, including Chinese women, have different menstrual and reproductive patterns as well as different lifestyle factors compared with women living in Western countries. At present, there is a lack of clear and comprehensive researches on the relationship between age at menarche, menopause, reproductive lifespan, and CVD risk in Chinese women. Based on a national representative cohort study, we investigate the associations between age at menarche and menopause, reproductive lifespan, and the CVD risk and examine the relationship in subgroups amongst Chinese postmenopausal women.

## Materials and Methods

### Study Design and Participants

**FIGURE 1:**
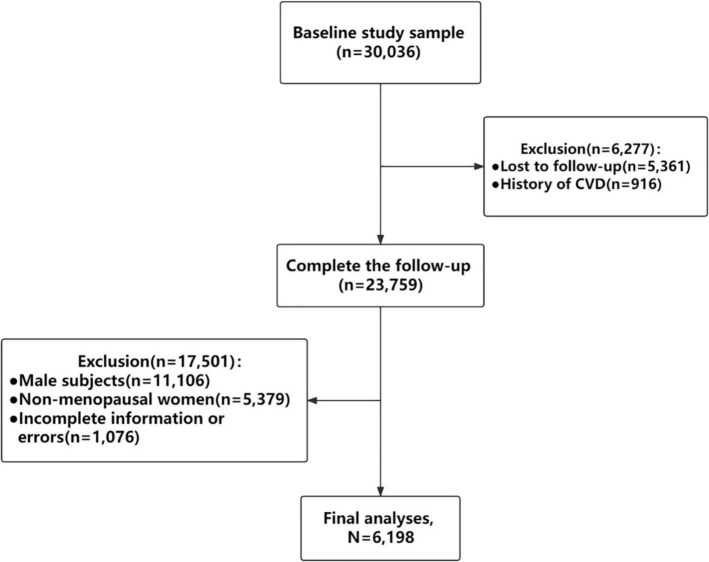
Flowchart of inclusion and exclusion of study participants. CVD, cardiovascular disease.

The Study on Prevalence and Key Technologies of Important Cardiovascular Diseases in China conducted from October 2012 to December 2015 was a large-scale representative CVD cross-sectional survey in China. A stratified multistage random sampling method was used to select about 500,000 nationally representative subjects aged ≥15 years from 262 districts and counties in 31 provinces of mainland China to investigate demographic characteristics, lifestyle risk factors, pharmacological treatment, female reproductive related characteristics and so on. In addition, the second sampling was carried out on the project. The selected districts/counties were divided into eastern, central and western region, and then stratified by urban and rural areas, 16 cities and 17 counties were selected by simple random sampling method. Random sampling was carried out in the above 33 districts/counties by stages. In the last stage, only respondents aged 35 and above were randomly selected from eligible regions to obtain blood samples for investigation of fasting plasma glucose (FPG), total cholesterol (TC), triglycerides (TG), low-density lipoprotein cholesterol (LDL-C), and high-density lipoprotein cholesterol (HDL-C) levels. Finally, those 30,036 individuals who completed the baseline survey were followed up in 2018–2019. 5,361 people lost to follow-up within the 5 years and the overall rate of follow-up was 82.15%. A total of 6,198 postmenopausal women were included in the final analysis after exclusions of 916 people with history of CVD, 11,106 male participants, 5,379 non-menopausal women and 1,076 postmenopausal women with incomplete information or errors (Figure 1).

The written informed consent was obtained from each participant. The Ethics Committee of Fuwai Hospital (Beijing, China) approved this study.

### Baseline Measurement and Data Collection

A standardized questionnaire was used to collect information on demographic characteristics, lifestyle risk factors, medical history, and characteristics related to female reproduction by well-trained interviewers in 2012–2015. Height and weight were measured using a standardized cartesian and an OMRON body fat and weight meter (V-body HBF-371, Omron, Japan) with participants wearing thin clothing and no shoes. Blood pressure of the participant was measured using an Omron HBP-1300 professional portable sphygmomanometer (Omron, Kyoto, Japan) on the right arm in the sitting position after resting for at least 5 min. Laboratory examinations were performed on venous blood samples collected after at least 8 h of fasting at night. FPG, TC, TG, LDL-C, and HDL-C levels were measured by a central core laboratory (Beijing Adicon Clinical Labora-tories, Inc., Beijing, China).

### Follow-Up and Outcome Measures

During 2018–2019, we tracked CVD events via interviewing participants or their proxies in-person or via telephone or mail questionnaires, and further examined medical records for reconfirmation. Participants’ CVD events were initially recorded by local investigators, and then hospital records were reviewed by the central adjudication committee of Fuwai Hospital (Beijing, China) to determine the final diagnosis. CVD events were defined as CHD, stroke, chronic heart failure, and death due to CVD. The criteria for stroke included non-fatal and fatal stroke (subarachnoid hemorrhage, ischemic stroke, intracerebral hemorrhage, and unspecified stroke). CHD was defined as non-fatal (including myocardial infarction, coronary artery bypass graft surgery, or percutaneous coronary intervention) and fatal CHD (such as fatal myocardial infarction and other coronary deaths).

### Variable Definition

At baseline, each participant was asked her age at the time of her first period, which was recorded as age at menarche. Menopausal status was defined based on the World Health Organization’s definition of menopause as the absence of menstruation for ≥12 months, and the age at which menopause occurred was recorded at baseline. The reproductive lifespan was defined as the interval between age at menarche and menopause.

Body mass index (BMI) was calculated by dividing weight (kg) by height squared (m^2^). Criteria for overweight and obese were defined as BMI between 24.0 and 27.9 kg/m^2^ and BMI ≥ 28.0 kg/m^2^, respectively (21). Alcohol drinking was defined as consuming an alcoholic beverage at least once per week in the past month. Smoking was defined as people who have smoked at least 20 packets of cigarettes in their lifetime and were still smoking (22). Diabetes was defined as fasting blood glucose ≥7 mmol/dL or was diagnosed with diabetes by a doctor or was prescribed hypoglycemic drugs within 2 weeks (23). According to the Guidelines for Dyslipidemia in China, TG ≥ 2.26mmol/L or TC ≥ 6.22mmol/L or HDL-C < 1.04 mmol/L or LDL-C ≥ 4.14 mmol/L or prior lipid-lowering medication or prior diagnosis of dyslipidemia (24). Have pharmacological treatment is defined as taking any one of anti-hypertensive, hypoglycemic, or anti-hyperlipidemia drugs. Comorbidity refers to any combination of two or more of three diseases: diabetes, hypertension and hypercholesterolemia following previous study (25). Parity was defined as the number of live babies a woman gives birth to, whether or not the baby dies after delivery (26).

### Statistical Analysis

Baseline characteristics of participants were presented as mean and standard deviation (SD) for normally distributed data or as a proportion for categorical data. The *t*-test, χ^2^ test, and variance analysis were used to compare variables between the different groups. Logistic linear regression model was used to explore the trend of variables. Multivariable Cox proportional hazard model was used to estimate Hazard ratios (HRs) and 95% confidence intervals (CIs) for CVD events associated with age at menarche (classified as ≤13, 14, 15, 16, and ≥17 years) with 15 years as the reference group, age at menopause (classified as ≤44, 45–46, 47–48, 49–50, and ≥51 years) with 47–48 years as the reference group, and reproductive lifespan (classified as ≤28, 29–31, 32–34, 35–37, and ≥38 years) with 32–34 years as the reference group.

This study further investigated the associations by additionally adjusting for age at recruitment (continuous), BMI (continuous), waist circumference (continuous), ethnicity (Han or other ethnicities), region (urban or rural), marital status (unmarried/widowed, married/cohabiting), education level (elementary or below, junior high school, high school or above), alcohol drinking (yes or no), smoking (yes or no), pharmacological treatment (yes or no), comorbidity (yes or no), family history of CVD (yes or no), ever pregnant (yes or no), contraceptive use (yes or no), breastfeeding experience (yes or no), and parity (0–1, 2, ≥3). We also examined the CVD risk by age at menarche and menopause and reproductive lifespan in subgroups of women defined by region, age at recruitment, marital status, BMI, education level, comorbidity, pharmacological treatment, alcohol drinking status, and family history of CVD, use of contraceptives, parity.

R 3.6.2 software were used to conduct analyses. The threshold of statistical significance was set at *p* < 0.05.

## Results

The characteristics of the study participants by age at menarche and menopause, and reproductive lifespan were listed in Tables 1–3, respectively. The mean (SD) age of study participants at recruitment was 63.6 (9.9) years, and the mean age at menarche and menopause, and reproductive lifespan were 15.9 (2.0), 48.7 (3.5), and 32.9 (3.9) years, respectively. The mean (SD) BMI, waist circumference, and parity were 24.8 (3.7) kg/m^2^, 83.9 (10.4) cm, and 3.0 (2.3), respectively. A minority of women were smokers (3.8%), alcohol drinkers (5.5%), and users of contraceptives (8.0%). Most women had previously pregnant (99.1%) and breastfeeding experience (96.2%). Women with earlier age at menarche had higher educational level, had lower BMI, waist circumference and parity, had higher percent of urban resident. Women with later age at menopause had higher educational level, BMI, and waist circumference, had higher percent of comorbidity, Women with lower reproductive lifespan had higher parity, had lower educational level, BMI and waist circumference, had lower percent of comorbidity.

**TABLE 1:** Characteristics of study participants by age at menarche.

	Age at menarche							
	
Characteristics	≤13	14	15	16	≥17	All	*P*_value_	*P*_trend_
Number (%)	754 (12.2)	860 (13.9)	1,086(17.5)	1,108(17.9)	2,390(38.6)	6,198(100)		
Age at recruitment								
Age, mean (SD)	62.0 (10.3)	62.3 (10.2)	63.9 (10.1)	63.2 (10.1)	64.7 (9.4)	63.6 (9.9)	<0.001	<0.001
<60, *n* (%)	358 (47.5)	398 (46.3)	407 (37.5)	443 (40.0)	744 (31.1)	2,350(38.0)	<0.001	<0.001
60–70, *n* (%)	206 (27.3)	232 (27.0)	338 (31.1)	370 (33.4)	942 (39.4)	2,088(33.7)		<0.001
≥70, *n* (%)	190 (25.2)	230 (26.7)	341 (31.4)	295 (26.6)	704 (29.5)	1,760(28.4)		0.064
**Baseline characteristics**								
Urban resident, *n* (%)	449 (59.6)	397 (46.2)	462 (42.5)	512 (46.2)	911 (38.1)	2,731(44.1)	<0.001	<0.001
Han ethnicity, *n* (%)	684 (90.7)	738 (85.8)	927 (85.4)	961 (86.7)	2,194(91.8)	5,504(88.8)	<0.001	0.001
Unmarried or widowed, n (%)	125 (16.6)	161 (18.7)	219 (20.2)	220 (20.0)	526 (22.1)	1,251(20.2)	0.016	
**BMI**								
BMI (kg/m^2^), mean (SD)	25.2 (3.6)	24.8 (3.6)	25.0 (3.8)	24.8 (3.7)	24.6 (3.6)	24.8 (3.7)	<0.001	<0.001
<24 kg/m^2^, *n* (%)	302 (40.1)	394 (45.8)	461 (42.5)	476 (43.0)	1,080(45.2)	2,713(43.8)	0.044	0.080
24–28 kg/m^2^, *n* (%)	295 (39.1)	309 (35.9)	404 (37.2)	438 (39.5)	911 (38.1)	2,357(38.0)		0.680
≥28 kg/m^2^, *n* (%)	157 (20.8)	157 (18.3)	221 (20.3)	194 (17.5)	399 (16.7)	1,128(18.2)		0.006
Waist circumference (cm), mean (SD)	84.5 (10.1)	83.9 (10.0)	84.1 (10.7)	84.2 (10.2)	83.5 (10.5)	83.9 (10.4)	0.131	0.035
Education level, *n* (%)								
Elementary or below	375 (49.7)	527 (61.3)	766 (70.5)	761 (68.7)	1,825(76.4)	4,254(68.6)	<0.001	<0.001
Junior high school	173 (23.0)	201 (23.4)	193 (17.8)	216 (19.5)	380 (15.9)	1,163(18.8)		<0.001
High school or above	206 (27.3)	132 (15.3)	127 (11.7)	131 (11.8)	185 (7.7)	781 (12.6)		<0.001
Alcohol drinking, *n* (%)	65 (8.6)	40 (4.7)	38 (3.5)	66 (6)	134 (5.6)	343 (5.5)	<0.001	0.229
Smoking, *n* (%)	31 (4.1)	32 (3.7)	36 (3.3)	54 (4.9)	85 (3.6)	238 (3.8)	0.203	0.952
Hypertension, *n* (%)	380 (50.4)	435 (50.6)	589 (54.2)	582 (52.5)	1,222(51.1)	3,208(51.8)	0.361	0.871
Diabetes, *n* (%)	123 (16.3)	108 (12.6)	135 (12.4)	139 (12.5)	290 (12.1)	795 (12.8)	0.050	0.021
Dyslipidemia, *n* (%)	288 (38.2)	267 (31)	404 (37.2)	397 (35.8)	814 (34.1)	2,170(35)	0.012	0.346
Comorbidity, *n* (%)	230 (30.5)	216 (25.1)	299 (27.5)	290 (26.2)	609 (25.5)	1,644(26.5)	0.062	0.040
Have pharmacological treatment, *n* (%)	278 (36.9)	285 (33.1)	412 (37.9)	381 (34.4)	830 (34.7)	2,186(35.3)	0.157	0.438
Ever pregnant, *n* (%)	750 (99.5)	853 (99.2)	1,076(99.1)	1,099(99.2)	2,366(99.0)	6,144(99.1)	0.807	0.276
Ever use of contraceptives, *n* (%)	67 (8.9)	78 (9.1)	85 (7.8)	92 (8.3)	175 (7.3)	497 (8.0)	0.433	0.086
Have breastfeeding experience, *n* (%)	693 (91.9)	825 (95.9)	1,038(95.6)	1,077(97.2)	2,330(97.5)	5,963(96.2)	<0.001	<0.001
**Parity**								
Parity, mean (SD)	2.6 (1.5)	2.9 (3.6)	3.0 (1.7)	3.0 (3.3)	3.1 (1.5)	3.0 (2.3)	0.345	<0.001
0–1, *n* (%)	199 (26.4)	150 (17.4)	169 (15.6)	156 (14.1)	239 (10)	913 (14.7)	<0.001	<0.001
2, *n* (%)	236 (31.3)	275 (32)	325 (29.9)	380 (34.3)	705 (29.5)	1,921(31)		0.354
≥3, *n* (%)	319 (42.3)	435 (50.6)	592 (54.5)	572 (51.6)	1,446(60.5)	3,364(54.3)		<0.001
Age at menopause, mean (SD)	48.4 (3.6)	48.5 (3.6)	48.9 (3.3)	48.7 (3.3)	48.9 (3.5)	48.8 (3.5)	0.002	<0.001
Reproductive lifespan, mean (SD)	35.8 (3.7)	34.5 (3.6)	33.9 (3.3)	32.7 (3.3)	31.0 (3.7)	32.9 (3.9)	<0.001	<0.001
Family history of CVD, n (%)	176 (23.3)	148 (17.2)	212 (19.5)	212 (19.1)	478 (20.0)	1,226(19.8)	0.041	0.526
**Follow-up event**								
CVD, *n* (%)	15 (2.0)	19 (2.2)	29 (2.7)	30 (2.7)	83 (3.5)	176 (2.8)	0.143	0.011

*Percentages were calculated based on women with complete information for that specific variable; SD, standard deviation; BMI, body mass index.*

**TABLE 2:** Characteristics of study participants by age at menopause.

	Age at menopause							
	
Characteristics	≤44	45–46	47–48	49–50	≥51	All	*P*_value_	*P*_trend_
Number (%)	647 (10.4)	761 (12.3)	1,127(18.2)	2,068(33.4)	1,595(25.7)	6,198(100.0)		
**Age at recruitment**								
Age, mean (SD)	61.9 (11.3)	63.0 (10.7)	63.0 (10.2)	64.7 (9.8)	63.7 (8.7)	63.6 (9.9)	<0.001	<0.001
<60, *n* (%)	278 (43.0)	302 (39.7)	441 (39.1)	704 (34)	625 (39.2)	2,350(37.9)	<0.001	0.015
60–70, *n* (%)	192 (29.7)	237 (31.1)	395 (35.0)	707 (34.2)	557 (34.9)	2,088(33.7)		0.011
≥70, *n* (%)	177 (27.4)	222 (29.2)	291 (25.8)	657 (31.8)	413 (25.9)	1,760(28.4)		0.956
**Baseline characteristics**								
Urban resident, *n* (%)	234 (36.2)	301 (39.6)	470 (41.7)	1,038(50.2)	688 (43.1)	2,731(44.1)	<0.001	<0.001
Han ethnicity, *n* (%)	555 (85.8)	655 (86.1)	981 (87)	1,854(89.7)	1,459(91.5)	5,504(88.8)	<0.001	0.001
Unmarried or widowed, n (%)	141 (21.8)	160 (21.1)	218 (19.4)	437 (21.2)	295 (18.6)	1,251(20.2)	0.231	0.134
**BMI**								
BMI (kg/m^2^), mean (SD)	24.4 (3.5)	24.8 (3.6)	24.6 (3.6)	24.8 (3.8)	25.0 (3.7)	24.8 (3.7)	0.001	<0.001
<24 kg/m^2^, *n* (%)	300 (46.4)	320 (42)	509 (45.2)	922 (44.6)	662 (41.5)	2,713(43.8)	0.050	0.128
24–28 kg/m^2^, *n* (%)	252 (38.9)	311 (40.9)	419 (37.2)	751 (36.3)	624 (39.1)	2,357(38)		0.486
≥28 kg/m^2^, *n* (%)	95 (14.7)	130 (17.1)	199 (17.7)	395 (19.1)	309 (19.4)	1,128(18.2)		0.005
Waist circumference (cm), mean (SD)	82.7 (10.2)	83.3 (10.1)	83.7 (10.2)	84.0 (10.7)	84.8 (10.2)	83.9 (10.4)	<0.001	<0.001
Education level, *n* (%)								
Elementary or below	466 (72.0)	533 (70.0)	752 (66.7)	1,426(69.0)	1,077(67.5)	4,254(68.6)	0.038	0.074
Junior high school	117 (18.1)	136 (17.9)	243 (21.6)	377 (18.2)	290 (18.2)	1,163(18.8)		0.722
High school or above	64 (9.9)	92 (12.1)	132 (11.7)	265 (12.8)	228 (14.3)	781 (12.6)		0.004
Alcohol drinking, *n* (%)	49 (7.6)	42 (5.5)	63 (5.6)	102 (4.9)	87 (5.5)	343 (5.5)	0.158	0.073
Smoking, *n* (%)	36 (5.6)	34 (4.5)	38 (3.4)	80 (3.9)	50 (3.1)	238 (3.8)	0.065	0.011
Hypertension, *n* (%)	298 (46.1)	373 (49.0)	556 (49.3)	1,100(53.2)	881 (55.2)	3,208(51.8)	<0.001	<0.001
Diabetes, *n* (%)	86 (13.3)	83 (10.9)	141 (12.5)	260 (12.6)	225 (14.1)	795 (12.8)	0.268	0.190
Dyslipidemia, *n* (%)	208 (32.1)	243 (31.9)	419 (37.2)	724 (35.0)	576 (36.1)	2,170(35.0)	0.070	0.047
Comorbidity, *n* (%)	147 (22.7)	187 (24.6)	301 (26.7)	545 (26.4)	464 (29.1)	1,644(26.5)	0.019	0.001
Have pharmacological treatment, *n* (%)	210 (32.5)	240 (31.5)	373 (33.1)	755 (36.5)	608 (38.1)	2,186(35.3)	0.003	<0.001
Ever pregnant, *n* (%)	643 (99.4)	755 (99.2)	1,115(98.9)	2,047(99)	1,584(99.3)	6,144(99.1)	0.711	0.907
Ever use of contraceptives, *n* (%)	44 (6.8)	69 (9.1)	107 (9.5)	137 (6.6)	140 (8.8)	497 (8.0)	0.014	0.908
Have breastfeeding experience, *n* (%)	619 (95.7)	725 (95.3)	1,090(96.7)	1,982(95.8)	1,547(97.0)	5,963(96.2)	0.159	0.102
**Parity, *n* (%)**								
Parity, mean (SD)	3.1 (4.1)	3.0 (1.6)	2.9 (1.5)	3.0 (2.6)	2.9 (1.5)	3.0 (2.3)	0.367	0.226
0–1	92 (14.2)	111 (14.6)	175 (15.5)	300 (14.5)	235 (14.7)	913 (14.7)	0.281	0.929
2	219 (33.8)	236 (31.0)	357 (31.7)	597 (28.9)	512 (32.1)	1,921(31.0)		0.326
≥3	336 (51.9)	414 (54.4)	595 (52.8)	1,171(56.6)	848 (53.2)	3,364(54.3)		0.396
Age at menarche, mean (SD)	15.8 (2.1)	15.7 (2.0)	15.8 (2.0)	16.0 (2.0)	16.0 (2.0)	15.9 (2.0)	0.001	<0.001
Reproductive lifespan, mean (SD)	25.9 (2.6)	29.7 (2.0)	31.8 (2.0)	33.7 (2.1)	36.8 (2.4)	32.9 (2.2)	<0.001	<0.001
Family history of CVD, n (%)	127 (19.6)	123 (16.2)	229 (20.3)	384 (18.6)	363 (22.8)	1,226(19.8)	0.002	<0.001
**Follow-up event**								
CVD, *n* (%)	24 (3.7)	17 (2.2)	31 (2.8)	54 (2.6)	50 (3.1)	176 (2.8)	0.446	0.907

*Percentages were calculated based on women with complete information for that specific variable; SD, standard deviation; BMI, body mass index.*

**TABLE 3:** Characteristics of study participants by reproductive lifespan.

Characteristics	Reproductive lifespan						*P*_value_	*P*_trend_
	
	≤28	29–31	32–34	35–37	≥38	All		
Number (%)	814 (13.1)	1,263(20.4)	1,897(30.6)	1,584(25.6)	640 (10.3)	6,198(100.0)		
Age at recruitment								
Age, mean (SD)	63.4 (10.8)	63.4 (10.2)	63.8 (10.1)	63.6 (9.4)	63.7 (8.7)	63.6 (9.9)	<0.001	0.429
<60, n (%)	296 (36.4)	449 (35.6)	716 (37.7)	620 (39.1)	269 (42.0)	2,350(37.9)	<0.001	0.005
60–70, n (%)	276 (33.9)	460 (36.4)	628 (33.1)	523 (33.0)	201 (31.4)	2,088(33.7)		0.077
≥70, *n* (%)	242 (29.7)	354 (28.0)	553 (29.2)	441 (27.8)	170 (26.6)	1,760(28.4)		0.240
**Baseline characteristics**								
Urban resident, *n* (%)	261 (32.1)	482 (38.2)	881 (46.4)	779 (49.2)	328 (51.2)	2,731(44.1)	<0.001	<0.001
Han ethnicity, *n* (%)	703 (86.4)	1,123(88.9)	1,673(88.2)	1,432(90.4)	573 (89.5)	5,504(88.8)	0.040	0.0129
Unmarried or widowed, *n* (%)	186 (22.9)	274 (21.8)	382 (20.2)	310 (19.6)	99 (15.5)	1,251(20.2)	0.006	0.001
**BMI**								
BMI (kg/m^2^), mean (SD)	24.3 (3.4)	24.6 (3.6)	24.8 (3.8)	24.9 (3.6)	25.3 (3.7)	24.8 (3.7)	<0.001	<0.001
<24 kg/m^2^, *n* (%)	375 (46.1)	578 (45.8)	823 (43.4)	681 (43.0)	256 (40.0)	2,713(43.8)	0.009	0.008
24–28 kg/m^2^, *n* (%)	328 (40.3)	465 (36.8)	716 (37.7)	600 (37.9)	248 (38.8)	2,357(38.0)		0.744
≥28 kg/m^2^, *n* (%)	111 (13.6)	220 (17.4)	358 (18.9)	303 (19.1)	136 (21.2)	1,128(18.2)		<0.001
Waist circumference (cm), mean (SD)	82.5 (10.0)	83.4 (10.4)	84.0 (10.7)	84.5 (10.1)	85.0 (10.2)	83.9 (10.3)	<0.001	<0.001
Education level, *n* (%)								
Elementary or below	644 (79.1)	895 (70.8)	1,314(69.3)	1,013(64.0)	388 (60.6)	4,254(68.6)	<0.001	<0.001
Junior high school	116 (14.3)	242 (19.2)	375 (19.7)	298 (18.8)	132 (20.6)	1,163(18.8)		0.010
High school or above	54 (6.6)	126 (10.0)	208 (11.0)	273 (17.2)	120 (18.8)	781 (12.6)		<0.001
Alcohol drinking, *n* (%)	51 (6.3)	74 (5.9)	101 (5.3)	70 (4.4)	47 (7.3)	343 (5.5)	0.061	0.622
Smoking, *n* (%)	41 (5.0)	58 (4.6)	67 (3.5)	51 (3.2)	21 (3.3)	238 (3.8)	0.100	0.010
Hypertension, *n* (%)	384 (47.2)	617 (48.9)	1,000(52.7)	842 (53.2)	365 (57.0)	3,208(51.8)	<0.001	<0.001
Diabetes, *n* (%)	100 (12.3)	139 (11.0)	232 (12.2)	227 (14.3)	97 (15.2)	795 (12.8)	0.028	0.006
Dyslipidemia, *n* (%)	258 (31.7)	418 (33.1)	694 (36.6)	563 (35.5)	237 (37.0)	2,170(35.0)	0.052	0.011
Comorbidity, *n* (%)	185 (22.7)	300 (23.8)	512 (27.0)	458 (28.9)	189 (29.5)	1,644(26.5)	0.001	<0.001
Have pharmacological treatment, *n* (%)	263 (32.3)	410 (32.5)	673 (35.5)	571 (36.0)	269 (42.0)	2,186(35.3)	<0.001	<0.001
Ever pregnant, *n* (%)	807 (99.1)	1,251(99)	1,877(98.9)	1,571(99.2)	638 (99.7)	6,144(99.1)	0.527	0.311
Ever use of contraceptives, *n* (%)	57 (7.0)	94 (7.4)	169 (8.9)	126 (8.0)	51 (8.0)	497 (8.0)	0.440	0.381
Have breastfeeding experience, *n* (%)	786 (96.6)	1,213(96.0)	1,842(97.1)	1,514(95.6)	608 (95.0)	5,963(96.2)	0.067	0.103
**Parity, *n* (%)**								
Parity, mean (SD)	3.1 (3.7)	3.0 (1.5)	3.1 (2.7)	2.9 (1.6)	2.7 (1.5)	3.0 (2.3)	0.490	0.002
0–1	103 (12.7)	141 (11.2)	256 (13.5)	296 (18.7)	117 (18.3)	913 (14.7)	<0.001	<0.001
2	248 (30.5)	413 (32.7)	588 (31.0)	457 (28.9)	215 (33.6)	1,921(31.0)		0.752
≥3	463 (56.9)	709 (56.1)	1,053(55.5)	831 (52.5)	308 (48.1)	3,364(54.3)		<0.001
Age at menarche, mean (SD)	17 (2.0)	16.8 (1.9)	16.1 (1.7)	15 (1.7)	14.1 (1.6)	15.9 (2.0)	<0.001	<0.001
Age at menopause, mean (SD)	42.9 (2.4)	47 (2.0)	49.1 (1.7)	50.8 (1.7)	53.3 (1.8)	48.7 (3.5)	<0.001	<0.001
Family history of CVD, *n* (%)	154 (18.9)	223 (17.7)	373 (19.7)	324 (20.5)	152 (23.8)	1,226(19.8)	0.029	0.006
**Follow-up event**								
CVD, *n* (%)	30 (3.7)	37 (2.9)	53 (2.8)	37 (2.3)	19 (3.0)	176 (2.8)	0.454	0.170

*Percentages were calculated based on women with complete information for that specific variable; SD, standard deviation; BMI, body mass index.*

**FIGURE 2:**
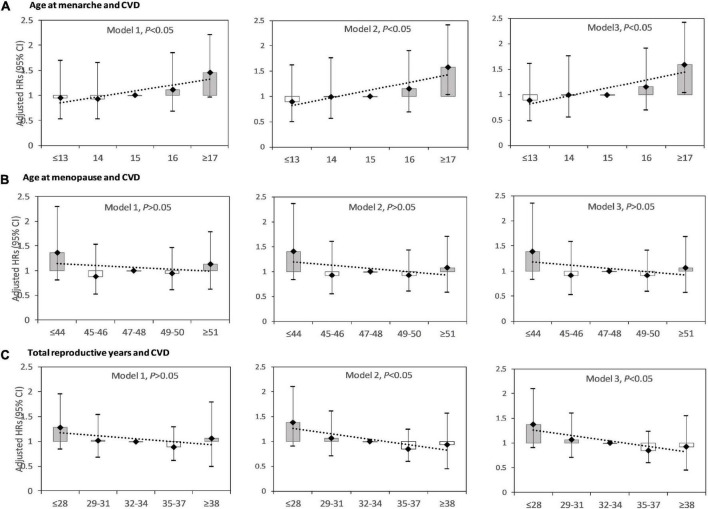
Hazard ratios (HRs) for cardiovascular disease (CVD) by age at menarche, age at menopause, and reproductive lifespan among postmenopausal women. **(A)** HRs for CVD by age at menarche; **(B)** HRs for CVD by age at menopause; **(C)** HRs for CVD by reproductive lifespan. Model 1: adjusted for age at recruitment. Model 2: model 1 plus region, ethnicity, marital status, body mass index, waist circumference, education level, alcohol drinking, smoking, comorbidity, pharmacological treatment, family history of CVD. Model 3: model 2 plus pregnant, contraceptive use status, and breastfeeding experience, parity.

Figure 2 showed the HRs for CVD events by age at menarche, age at menopause, and reproductive lifespan amongst postmenopausal women. In model 1, the association between age at menarche and CVD was significant, and the association between age menopause, reproductive lifespan and CVD was not significant. In model 2 and 3, the association between age at menarche, reproductive lifespan and CVD was significant, and the association between age menopause and CVD was not significant. Model 3 showed that for those with age at menarche ≤ 13, 14, 15 (reference), 16, and ≥ 17 years, the HRs (95%CIs) were respectively 0.887 (0.489–1.609), 0.993 (0.560–1.762) 1.000 (reference), 1.157 (0.699–1.915), and 1.589 (1.041–2.424), for those with age at menopause ≤44, 45–46, 47–48 (reference), 49–50, and ≥51 years, the HRs (95%CIs) were respectively 1.388n(0.821–2.346), 0.861 (0.482–1.537), 1.000 (reference), 0.909 (0.587–1.405), and 1.111. (0.716–1.726), for those with reproductive lifespan ≤28, 29–31, 32–34 (reference), 35–37, and ≥38, the HRs (95%CIs) were respectively 1.368 (0.896–2.090), 1.059 (0.700–1.602), 1.000 (reference), 0.746 (0.492–1.132), and 0.926 (0.538–1.549). The highest risk was seen in those with menarche at age ≥17 years, and those with reproductive lifespan ≤28 years.

Age at menarche was positively associated with CVD events, with an adjusted HR (95%CI) of 1.106 (1.047–1.167) per year. For every 1-year increase in age at menarche, the risk of CVD increased by 10.6%. The association between age at menopause and risk of CVD was not significant, with an adjusted HR (95%CI) of 0.982 (0.943–1.024) per year. Reproductive lifespan was negatively associated with CVD events, with an adjusted HR (95%CI) of 0.962 (0.929–0.996) per year. For every 1-year increase in reproductive lifespan, the risk of CVD was reduced by 3.8%.

**FIGURE 3:**
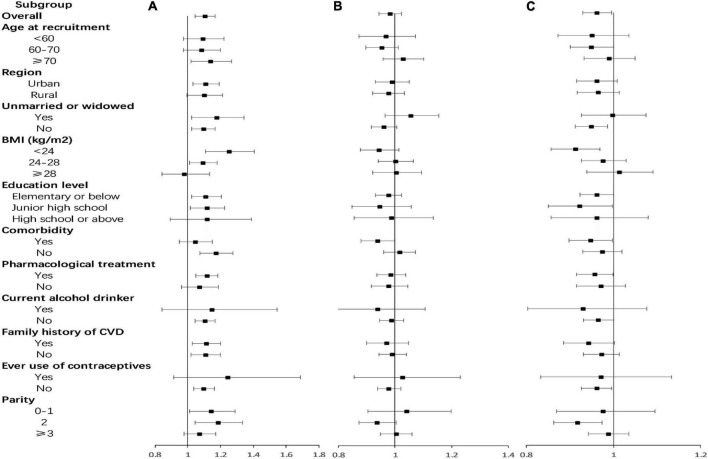
Adjusted hazard ratios (HRs) for cardiovascular disease (CVD) per year by age at menarche, age at menopause, and reproductive lifespan within various subgroups. **(A)** Age at menarche; **(B)** age at menopause; **(C)** reproductive lifespan. Analyses were adjusted for age at recruitment, region, ethnicity, marital status, body mass index, waist circumference, education level, alcohol drinking, smoking, comorbidity, pharmacological treatment, family history of CVD, pregnant, contraceptive use status, and breastfeeding experience, parity.

Subgroup analyses were also conducted according to region, age at recruitment, marital status, BMI, education level, pharmacological treatment, comorbidity, alcohol drinking status, use of contraceptives, parity, and family history of CVD (Figure 3). There was a positive association between age at menarche and risk of CVD events in postmenopausal women within the following subgroups: living in urban areas, 70 years old and above, low BMI, low education level, have no comorbidity, have pharmacological treatment, non-drinkers, no previous contraceptive use, and 0–2 live births. There was a negative association between reproductive lifespan and risk of CVD events among the following subgroups: married, 60–70 years old, low BMI, low education level, have comorbidity, have pharmacological treatment, non-drinkers, no previous contraceptive use, and 2 live births. There was a negative association between age at menopause and CVD risk in postmenopausal women with comorbidity, with an adjusted HR (95%CI) of 0.938 (0.880–0.999) per year. The risk of CVD reduced by 6.2% for every 1-year increase in age at menopause in postmenopausal women with comorbidity.

## Discussion

To our knowledge, this was the first large prospective study that comprehensively investigated the associations between age at menarche and menopause, reproductive lifespan, and risk of CVD events in Chinese postmenopausal women using a nationally representative sample and evaluating multiple subgroups. Based on 6,198 Chinese postmenopausal women without prior history of CVD, we found that late age at menarche and shorter reproductive span is significantly associated with increased risk in CVD events among women. These associations also appeared to be similar amongst subgroups. There was also a negative association between age at menopause and CVD risk in postmenopausal women with comorbidity. For every 1-year increase in age at menarche, the risk of CVD increased by 10.6%, and for every 1-year increase in reproductive lifespan, the risk of CVD reduced by 3.8%. The risk of CVD reduced by 6.2% for every 1-year increase in age at menopause in women with comorbidity.

Several studies have previously investigated the relationship of age of menarche and CVD events, but reported inconsistent findings. A systematic review revealed that for Caucasian populations, eight out of twelve studies show a generally linear association between a decrease in age at menarche with an increase in CVD risk (11). But the UK Million Women Study showed a significant U-shaped relationship between age at menarche and the outcomes CHD (6). This discrepancy may be due in part to small sample sizes, geographic limits to one city or province, or only rural areas, age or ethnic differences, or residual confounding from unmeasured or other potential risk factors. The results from studies with Asian populations are inconclusive, with either early or late menarche increasing the CVD risk. One cohort study reported an increased risk of ischemic stroke for women with an age at menarche ≤13 years compared to those with 15 years, but the participants were selected from a rural town in northern Japan and therefore not representativeness of the general population (27). The same U-shape between age at menarche and CVD risk was found in data from the Chinese Kadoori Biobank (CKB), but the associations seemed to differ by birth cohort (28). A case-control study reported that higher age at menarche was associated with an increased ischemic stroke risk, which was consistent with our study (29).

Some mechanisms may explain the positive correlation between age of menarche and risk of CVD events in our results. Studies have shown that late menarche is associated with low estrogen levels (30, 31). Estrogen affects the elasticity of blood vessels and regulates the level of metabolic mediators such as lipids, inflammatory markers and the coagulation system (4). Estrogen has a protective effect on CVD in women by stimulating endothelial nitric oxide synthase, which mediates vasodilation and maintains cardiovascular health (32, 33). Women with early age at menarche are more likely to have higher levels of ovarian hormone, which can protect women from hypertension and atherosclerosis (34). Several studies agree about the different impact of testosterone on males and females (35, 36). Low endogen levels of this hormone in males can be associated with CVD and coronary stenosis severity. High levels of circulating androgens in women appear to be associated with higher cardiovascular risk (37, 38). In addition, age at menarche is associated with the higher adiposity both in childhood and in adulthood (39, 40). Adiposity indicators mediated the relationship between age at menarche and blood pressure in women. Adiposity is a risk factor of CVD, so the association between age at menarche and CVD could a result of higher estrogen levels or higher adiposity in early maturing women (40).

Additionally, the relationships between age at menopause and CVD risk in previous research are contradictory. Two meta-analysis indicated women with early menopausal age have a higher risk of incident CVD (16, 41). However, the result might have introduced some heterogeneity or has certain limitations about extrapolation due to the limited racial population included. Results of UK Biobank on early natural menopause were associated with a increased risk for a composite of CVD may have a “healthy participant” selection bias (42). The following studies are consistent with our findings on no convincing relationship between early menopausal age and cardiovascular disease (12, 43–47). Differences in population and study design and the influence of confounding factors may be the main reasons for the inconsistent results.

Persuasive evidence demonstrated that bilateral oophorectomy in premenopausal women increases the risk of CHD unless exogenous hormones are administered (48). However, there is no convincing evidence to support the hypothesis that natural menopause is a risk factor for CHD. When women with natural, surgical, and medicated menopause were analyzed together, the risk increased significantly with decreasing age at menopause, but this inverse association was limited to current non-smokers and not statistically significant amongst women with natural menopause (49). In a previous report from the Nurses’ Health Study, with further adjustment for smoking and age the relative risk of CHD in postmenopausal women diminished from significantly elevated to insignificant (48). These results might reflect the need to closely control the residual confounding factors of smoking and age, which have been adjusted for in our study. Furthermore, we found that there was a negative association between age at menopausal and CVD risk in postmenopausal women with comorbidity. As far as we know, diabetes, hypertension and dyslipidemia are independent risk factors for CVD events and show a additive association with the risk of CVD (50). Our finding suggests that this association may be more striking to particularly attention in postmenopausal women.

Previous studies investigating the association between reproductive lifespan and CVD risk were inconsistent. A systematic review and meta-analysis found that women with a shorter reproductive lifespan was associated with a higher risk of CVD events, with a 31% increased risk of stroke in particular (19). The Nurse’s Health Study found that the CVD risk was 25% higher amongst those with <30 years of reproductive lifespan compared to 34–37 years (51). One cross-sectional study from the United States showed that each yearly increase in reproductive lifespan was associated with 3% reduction in the risk for overall CVD and stroke events (20). The findings from this and other studies were consistent for CHD (34, 51, 52). A cohort study from the Women’s Health Initiative manifested that a shorter total reproductive duration in postmenopausal women resulted in a modestly increased risk of any heart failure (53). In Asia, the study of CKB found that total reproductive years were inversely associated with risks of both fatal and non-fatal CVD events, with 1.4% lower risk of CVD events death per each additional reproductive year (52). For stroke mortality, a large prospective study from Japan showed a decreased risk for longer reproductive lifespan (54). The above relationship between reproductive lifespan and CVD risk is consistent with our current study. However, some studies have found that there is no statistically significant association between the number of reproductive years and CVD mortality (9, 46, 55, 56). In addition, Jung et al. found a U-shaped association between reproductive lifespan and ischemic heart disease; the risk of which increased in both reproductive lifespan <30 and 40 years when 36–39 years were used as a reference (34). In one prospective study, it was significantly associated with 5% higher mortality for every additional year of reproductive lifespan (57).

Timing of menarche and menopause were correlated in that women who experienced early menarche were at higher risk of early menopause (51). In addition, reproductive lifespan can serve as a simple way to estimate estrogen exposure (51). Therefore, the underlying mechanism linking a longer reproductive lifespan with reduced risk of CVD events can be attributed to prolonged exposure to endogenous estrogen, which is accordant with our previous explanatory mechanism. These findings suggest that reproductive factors may play an important role in maintaining and improving cardiovascular health, which can be used to assess populations with poor cardiovascular health for targeted interventions.

## Strengths and Limitations

Important strengths of our study are the prospective design and nationally representative sample. The study has several other strengths, including the standardized approaches and stringent quality control measures taken to ensure data quality and reliability. We adjusted for potential risk factors for CVD and conducted a detailed subgroup analysis to improve the reliability of our results, which allowed us to simultaneously adjust for potential confounders and reliably assess the associations.

Several limitations need to be taken into account. First, CVD is composed of multiple diseases, so it is unclear whether role in our study is specific for certain CVD. Second, age at menarche is known to vary by ethnicity, with girls of African and Asian descent experiencing earlier menarche than Caucasian girls (58, 59). However, it is unknown whether ethnicity plays a role in the association between age at menarche and CVD events. Furthermore, our study participants were Chinese women, which minimized the confounding effects by ethnic background, but might reduce the generalization of our results to other ethnic groups. Thirdly, ages at the time of menarche and menopause were self-reported which raises the possibility of misclassification due to recall bias. However, previous studies have reported that the recall of age at menarche (60, 61) and menopause is relatively accurate (62, 63). Therefore, self-reported age at menarche and menopause were reasonably valid and reproducible. If recall bias existed, it would be non-differential, tending to attenuate the real strengths of the associations that we observed. Lastly, although we have adjusted for a comprehensive range of potential confounders, the possibility of residual confounding from other known or unknown risk factors cannot be completely ruled out.

## Conclusion

Based on 6,198 Chinese postmenopausal women who had no prior history of CVD, we found that late age at menarche and shorter reproductive span are both significantly associated with increased risk of CVD events. These associations also appeared to be similar amongst subgroups. In addition, there was a negative association between age at menopause and CVD risk in postmenopausal women with comorbidity. Our findings have important public health implications for early detection and timely implementation of appropriate interventions in women at high risk of CVD.

## Data Availability Statement

Data from this study cannot be used publicly. Requests to access these datasets should be directed to the corresponding author.

## Ethics Statement

The studies involving human participants were reviewed and approved by the Ethics Committee of Fuwai Hospital (Beijing, China). The patients/participants provided their written informed consent to participate in this study.

## Author Contributions

LC: methodology, formal analysis, software, and writing – original draft preparation partly. ZH: conceptualization and writing – original draft preparation partly. XW and YS: investigation and writing – original draft preparation partly. ZC, LZ, and CZ: data curation, formal analysis, and software. HZ, XC, YT, JC, RG, and YH: investigation. JV: language polishing. ZW: conceptualization, funding acquisition, and writing—reviewing and editing. All authors contributed to the article and approved the submitted version.

## Abbreviations

CVD: cardiovascular disease
CHD: coronary heart disease
BMI: body mass index
SD: standard deviation
HRs: hazard ratios
CIs: confidence intervals
CKB: Chinese Kadoori Biobank.
